# First generation anti-CD19 chimeric antigen receptor-modified T cells for management of B cell malignances: initial analysis of an ongoing Phase I clinical trial

**DOI:** 10.1186/2051-1426-2-S3-P12

**Published:** 2014-11-06

**Authors:** Manon Evans, Ryan Guest, Dominic Rothwell, Debbie Burt, Natalia Kirillova, Jennifer Haughton, Shien Chow, Fiona Thistlethwaite, David Gilham, Robert Hawkins

**Affiliations:** 1 Department of Medical Oncology, Christie NHS Foundation Trust, Manchester, United Kingdom; 2 Cellular Therapeutics Limited, Manchester, United Kingdom; 3 Clinical Immune and Molecular Monitoring Laboratory, Clinical and Experimental Pharmacology Group, Cancer Research UK Manchester Institute, Manchester, United Kingdom; 4 Clinical and Experimental Immunotherapy Group, Institute of Cancer Sciences, Manchester Academic Healthcare Science Centre, Manchester, United Kingdom

## Introduction

Management of advanced B cell malignancies refractory to standard chemotherapy is challenging with sub-optimal results. Recent clinical reports of durable, objective responses from adoptive transfer of anti-CD19 chimeric antigen receptor (CAR) T cells have accentuated the potential of this therapy.

Here we report the preliminary results of an on-going Phase I clinical trial at our Institution.

## Methods

This is a single centre, open label, dose escalation, Phase I study of adoptive transfer of autologous T cells expressing a CD19-specific first generation CAR (aCD19z) with pre-conditioning chemotherapy and intravenous interleukin-2 (IL2), in patients with pre-treated CD19-positive malignancy.

We report data on 2 cohorts: Cohort 1 (4 patients) received 1x10^9 ^aCD19z T-cells and Cohort 2 (planned 4 patients) 1x10^10. ^Both cohorts received 100,000 u/kg of IL2.

## Results

To date, 6 patients have successfully completed treatment. All patients tolerated treatment well, and experienced anticipated transient grade1-2 toxicities attributable to pre-conditioning chemotherapy and IL2. 5 patients developed short lived but significant Grade 4 neutropenia and thrombocytopenia.

4 of 5 patients evaluable to date achieved at least stable disease as best response at 6 weeks post aCD19z T cell infusion, with 1 patient maintaining response at 400+ days. 1 patient achieved a very good partial response with a 65% reduction in disease burden. 2 patients died of disease progression (1 of Central Nervous (CN) progression only, not present at baseline); 2 patients died of viral infection over 400 days post infusion; 1 patient with disease control maintained. No patients died of treatment-related complications.

Quantitative polymerase chain reaction (qPCR) analysis of peripheral blood samples detected aCD19z T cells in both cohorts. Levels peaked at days 4-7 post aCD19z T cell infusion (cohort 1 peak 30% of total cells, cohort 2 (results available to date) peak 25%) before falling to lower levels. All patients revealed persisting low frequency levels (<1%) at week 6; 1 patient at up to 50 weeks. 1 patient received a further course of low dose IL2 at week 6 resulting in a transient increase in zCD19z T cell levels.

All patients demonstrated a significant reduction in peripheral CD19+ T cell numbers post aCD19z T cell infusion, with most substantial results seen in cohort 2 where suppression was seen lasting into week 8 (prior to CN progression).

## Discussion

Our data contributes to the encouraging growing body of evidence on antiCD19-specific CAR T cells, suggesting significant clinical responses and sustained persistence. Updated results and immune data will be presented.

## Registration Details

http://www.clinicaltrials.gov NCT01493453

## Consent

Written informed consent was obtained from the patient for publication of this abstract and any accompanying images. A copy of the written consent is available for review by the Editor of this journal.

